# Assessment of an Intervention to Reduce Aspirin Prescribing for Patients Receiving Warfarin for Anticoagulation

**DOI:** 10.1001/jamanetworkopen.2022.31973

**Published:** 2022-09-19

**Authors:** Jordan K. Schaefer, Josh Errickson, Xiaokui Gu, Tina Alexandris-Souphis, Mona A. Ali, Brian Haymart, Scott Kaatz, Eva Kline-Rogers, Jay H. Kozlowski, Gregory D. Krol, Vinay Shah, Suman L. Sood, James B. Froehlich, Geoffrey D. Barnes

**Affiliations:** 1 Division of Hematology/Oncology, Department of Internal Medicine, University of Michigan, Ann Arbor; 2 Consulting for Statistics, Computing, & Analytics Research, University of Michigan, Ann Arbor; 3 Division of Cardiovascular Medicine, Department of Internal Medicine, University of Michigan, Ann Arbor; 4 Department of Heart and Vascular Services, Beaumont Hospital, Royal Oak, Michigan; 5 Division of Hospital Medicine, Henry Ford Hospital, Detroit, Michigan; 6 Huron Valley Sinai Hospital, Commerce Township, Michigan; 7 Department of Internal Medicine, Henry Ford Hospital, Detroit, Michigan

## Abstract

**Importance:**

For some patients receiving warfarin, adding aspirin (acetylsalicylic acid) increases bleeding risk with unclear treatment benefit. Reducing excess aspirin use could be associated with improved clinical outcomes.

**Objective:**

To assess changes in aspirin use, bleeding, and thrombosis event rates among patients treated with warfarin.

**Design, Setting, and Participants:**

This pre-post observational quality improvement study was conducted from January 1, 2010, to December 31, 2019, at a 6-center quality improvement collaborative in Michigan among 6738 adults taking warfarin for atrial fibrillation and/or venous thromboembolism without an apparent indication for concomitant aspirin. Statistical analysis was conducted from November 26, 2020, to June 14, 2021.

**Intervention:**

Primary care professionals for patients taking aspirin were asked whether an ongoing combination aspirin and warfarin treatment was indicated. If not, then aspirin was discontinued with the approval of the managing clinician.

**Main Outcomes and Measures:**

Outcomes were assessed before and after intervention for the primary analysis and before and after 24 months before the intervention (when rates of aspirin use first began to decrease) for the secondary analysis. Outcomes included the rate of aspirin use, bleeding, and thrombotic outcomes. An interrupted time series analysis assessed cumulative monthly event rates over time.

**Results:**

A total of 6738 patients treated with warfarin (3160 men [46.9%]; mean [SD] age, 62.8 [16.2] years) were followed up for a median of 6.7 months (IQR, 3.2-19.3 months). Aspirin use decreased slightly from a baseline mean use of 29.4% (95% CI, 28.9%-29.9%) to 27.1% (95% CI, 26.1%-28.0%) during the 24 months before the intervention (*P* < .001 for slope before and after 24 months before the intervention) with an accelerated decrease after the intervention (mean aspirin use, 15.7%; 95% CI, 14.8%-16.8%; *P* = .001 for slope before and after intervention). In the primary analysis, the intervention was associated with a significant decrease in major bleeding events per month (preintervention, 0.31%; 95% CI, 0.27%-0.34%; postintervention, 0.21%; 95% CI, 0.14%-0.28%; *P* = .03 for difference in slope before and after intervention). No change was observed in mean percentage of patients having a thrombotic event from before to after the intervention (0.21% vs 0.24%; *P* = .34 for difference in slope). In the secondary analysis, reducing aspirin use (starting 24 months before the intervention) was associated with decreases in mean percentage of patients having any bleeding event (2.3% vs 1.5%; *P* = .02 for change in slope before and after 24 months before the intervention), mean percentage of patients having a major bleeding event (0.31% vs 0.25%; *P* = .001 for change in slope before and after 24 months before the intervention), and mean percentage of patients with an emergency department visit for bleeding (0.99% vs 0.67%; *P* = .04 for change in slope before and after 24 months before the intervention), with no change in mean percentage of patients with a thrombotic event (0.20% vs 0.23%; *P* = .36 for change in slope before and after 24 months before the intervention).

**Conclusions and Relevance:**

This quality improvement intervention was associated with an acceleration of a preexisting decrease in aspirin use among patients taking warfarin for atrial fibrillation and/or venous thromboembolism without a clear indication for aspirin therapy. Reductions in aspirin use were associated with reduced bleeding. This study suggests that an anticoagulation clinic–based aspirin deimplementation intervention can improve guideline-concordant aspirin use.

## Key Points

**Question:**

Is it possible to reduce excess aspirin (acetylsalicylic acid) use among patients treated with warfarin, and is reducing excess aspirin use associated with improved clinical outcomes?

**Findings:**

This multicenter quality improvement study of 6738 adults taking warfarin for atrial fibrillation and/or venous thromboembolism without an apparent indication for concomitant aspirin found that an anticoagulation clinic–based aspirin deimplementation intervention was associated with a significant acceleration of a preexisting decrease in excess aspirin use. Reducing aspirin use was associated with significantly less bleeding and health care use; no increase in thrombotic outcomes was observed.

**Meaning:**

This study suggests that it is possible to reduce aspirin use without a clear indication and that this effort may be associated with improved clinical outcomes.

## Teaser

This quality improvement study assesses changes in aspirin use, bleeding, and thrombosis event rates among patients treated with warfarin for atrial fibrillation and/or venous thromboembolism without an apparent indication for concomitant aspirin.

## Introduction

Aspirin (acetylsalicylic acid) is used for the primary prevention of coronary artery disease,^1,2^ for stable ischemic heart disease,^3^ for peripheral arterial disease,^4,5^ and/or for the secondary prevention of stroke after a noncardioembolic stroke or transient ischemic attack.^6,7^ Aspirin is appropriately combined with warfarin for some patients with atrial fibrillation or venous thromboembolism after acute coronary syndromes or percutaneous coronary interventions^8,9^ and for some patients with mechanical heart valves.^10,11,12^ For most other patients, evidence suggests that a combination of warfarin and aspirin therapy likely does more harm than good by increasing bleeding events without a clear reduction in thrombotic outcomes.^13,14,15,16,17,18,19^ Combination therapy with warfarin plus aspirin is estimated to result in a 1.5- to 2.0-fold risk of major bleeding compared with warfarin alone.^18^ For every 1000 patients, combination therapy may add 10 to 20 major bleeding events and 1 to 2 deaths per year compared with warfarin monotherapy.^19^ Many patients appear to be receiving aspirin even when the anticipated risk exceeds the benefit.

Recognizing the potential harm of combination warfarin and aspirin therapy, guidelines for atrial fibrillation with stable coronary artery disease,^9^ stroke,^6^ peripheral arterial disease,^4,5^ and aspirin for primary prevention^20^ suggest that warfarin monotherapy may be sufficient for most patients. A recent study confirmed the adverse effects of warfarin plus aspirin in a large registry-based cohort of patients receiving warfarin without a history of valve replacement or recent myocardial infarction who were followed up by the Michigan Anticoagulation Quality Improvement Initiative (MAQI^2^).^13^ Patients taking aspirin plus warfarin had significantly higher bleeding rates but a similar rate of thrombotic outcomes.

Given the high rate of inappropriate aspirin use and associated harms among patients treated with warfarin, each of the 6 clinical sites of the MAQI^2^ implemented a common intervention to reduce high-risk aspirin use. We sought to evaluate the preintervention and postintervention proportion of patients receiving aspirin without a clear indication. We also sought to evaluate the association of the intervention with clinical outcomes.

## Methods

### Study Design and Participants

The MAQI^2^ is a collaborative of 6 outpatient anticoagulation clinics throughout Michigan that includes both academic and community practices^21^; all forms of health insurance are accepted. These anticoagulation clinics represent rural and urban practices, with patient censuses ranging from hundreds to more than 5000 (eTable in the Supplement). Each participating site used a tailored screening process to identify adults receiving warfarin for atrial fibrillation and/or venous thromboembolism who were also receiving concomitant aspirin that may have been inappropriate. Potential inappropriate aspirin use was assessed based on an agreed-on set of criteria. Specifically, patients targeted for review of their ongoing aspirin use were adults without a history of coronary artery disease, myocardial infarction, any percutaneous coronary intervention, coronary artery bypass grafting, peripheral arterial disease, mechanical valve replacement, or use of left ventricular assist devices who were taking warfarin for atrial fibrillation or venous thromboembolism. Sites were encouraged to further limit patients targeted for review based on their institutional practice patterns (eg, some sites did not include patients with a history of stroke, heart transplant, or antiphospholipid syndrome). Site-specific screening processes are summarized in the eTable in the Supplement. This study follows the Standards for Quality Improvement Reporting Excellence (SQUIRE) reporting guideline and was approved by the institutional review board at all participating centers before data collection. A waiver of informed consent was granted by the institutional review boards at each participating center and the coordinating center because this study was a quality improvement project.

If screening found that a patient’s indication for aspirin use was unclear or potentially inappropriate, communication with the patient’s primary care physician or managing specialist ensued to alert them to their patient’s use of aspirin and discuss the need for therapy. All patient management decisions were deferred to the managing physician, but input was provided by the anticoagulation clinic staff. To allow for local tailoring of the intervention, the various sites differed in the personnel carrying out the intervention, how technology was used, and how clinicians were contacted.

The quality improvement interventions were enacted between October 1, 2017, and June 30, 2018; all analyses used site-specific dates of the intervention to compare preintervention and postintervention data. We referred to the period 96 months prior to intervention to 24 months prior to intervention as the historical period, the period from 24 months prior to the intervention until the intervention as the preintervention period, and the 24 months after the intervention as the postintervention period. Data used for this analysis were collected from January 1, 2010, through December 31, 2019.

### Data Collection and Outcome Measures

Patients were followed up from the time of MAQI^2^ enrollment until they were discharged from the anticoagulation clinic, they were lost to follow-up, the end of the study period, or death. Given the broad catchment of our hospital network with comprehensive follow-up, we do not think that the study findings were associated with patients entering or leaving the registry. Data collection was performed by trained abstractors using standardized data collection forms. Through combined use of wide-ranging validation rules during data entry and an automated program that identifies missing information and prompts for completion and correction, there were no missing data in the important variables used in the analysis. Body mass index could not be calculated for all patients owing to missing data in the primary medical records, but body mass index was not used in the analysis of aspirin use or clinical outcomes. Random medical record audits were performed by the coordinating center to ensure that the abstracted data matched the primary electronic medical records.^21^

Data collected at study enrollment included patient demographic characteristics, comorbidities, bleeding and thrombosis risk factors, histories of bleeding or thrombosis, and concomitant medications (including antiplatelet therapies other than aspirin). The HAS-BLED (hypertension, abnormal kidney or liver function, stroke, bleeding history or predisposition, labile international normalized ratio, elderly, drugs or alcohol concomitantly) score^22^ and the CHA_2_DS_2_-VASc (congestive heart failure, hypertension, age ≥75 years, diabetes, prior stroke or transient ischemic attack, vascular disease, age 65-74 years, and sex category [female]) risk score^23^ were calculated for each patient at the time of study enrollment, with the HAS-BLED score modified to exclude the labile international normalized ratio.

Indications for aspirin use were assessed at enrollment for the preintervention cohort and assessed at either enrollment or the first follow-up after the implementation of the intervention for the postintervention cohort. Our primary outcome was the rate of inappropriate aspirin use over time. Inappropriate aspirin use was assessed by each site based on their assessment of patients who might benefit from review by their physician.

Our secondary outcomes were rates of any bleeding, major bleeding as defined by the International Society on Thrombosis and Haemostasis,^24^ nonmajor bleeding (defined as any bleeding that did not meet the definition of major bleeding), and thrombosis. Thrombotic outcomes included ischemic or embolic stroke, transient ischemic attack, pulmonary embolism, deep vein thrombosis, intracardiac thrombus, or other or unknown clot. We also assessed rates of emergency department visits and hospitalizations related to bleeding.

### Statistical Analysis

Statistical analysis was conducted from November 26, 2020, to June 14, 2021. Initial review of aspirin use demonstrated a decrease starting approximately 24 months prior to the intervention. Therefore, in addition to our primary analysis comparing outcomes before and after the date of the aspirin-deprescribing intervention, we also conducted a secondary analysis comparing outcomes before and after the initial time of aspirin decrease (24 months prior to the aspirin-deprescribing intervention).

Given the dynamic nature of clinic-level patient volumes, risk profiles over time, and medication use, we used monthly aggregated data as the unit of analysis. Thus, the outcomes represent the percentage of patients treated with warfarin who experienced an event each month.

To examine the changing percentage of patients taking aspirin without a clear indication who experienced an outcome event, we conducted interrupted time series analyses by way of a linear regression model. This model contained binary variables indicating before or after the intervention representing the interruption. From this model, we could then estimate the trajectory of the percentage of patients in the preintervention and postintervention periods separately, to test whether those trajectories differed. A secondary interrupted time series analysis used 24 months prior to the aspirin-deprescribing intervention (the time when aspirin use began to decrease) as the interruption time point. Given the nature of monthly aggregated data, comparisons of event rates were not adjusted for patient-level data. A 2-sided *P* < .05 was considered statistically significant for all comparisons. All statistical analyses were performed using SAS, version 9.4 (SAS Institute Inc) and Stata, version 16 (StataCorp LLC).

## Results

A total of 6738 patients receiving warfarin without an indication for aspirin were followed up by the MAQI^2^ (3160 men [46.9%; 95% CI, 45.7%-48.1%]; mean [SD] age, 62.8 [16.2] years) for a median of 6.7 months (IQR, 3.2-19.3 months). Most patients (3714 [55.1%]) received warfarin for anticoagulation for venous thromboembolic disease (Table).

**Table.** Characteristics of Study Cohort

Characteristic	Patients, No. (%) (N = 6738)
Sex	
Male	3160 (46.9)
Female	3578 (53.1)
Age at enrollment, y	
Mean (SD)	62.8 (16.2)
Median (IQR)	64.3 (52.2-75.0)
Weight <50 kg	177/6483 (2.7)
BMI >30	3062/6288 (48.7)
Alcohol or drug use	329 (4.9)
Tobacco use	
Former	1754 (26.0)
Current	569 (8.4)
HAS-BLED score at enrollment	
Mean (SD)^a^	2.0 (1.3)
Median (IQR)	2.0 (1.0-3.0)
CCI at enrollment	
Mean (SD)	3.2 (1.9)
Median (IQR)	3.0 (2.0-5.0)
CHA_2_DS_2_-VASc risk score at enrollment, mean (SD)	
Mean (SD)	2.2 (1.5)
Median (IQR)	2.0 (1.0-3.0)
Indication at enrollment	
Atrial fibrillation or atrial flutter only	2955 (43.9)
Deep vein thrombosis or pulmonary embolism only	3714 (55.1)
Both	69 (1.0)
Comorbidities at enrollment	
Cancer	1355 (20.1)
Congestive heart failure	753 (11.2)
Chronic liver disease	149 (2.2)
Chronic kidney disease	736 (10.9)
Diabetes	1359 (20.2)
History of falls	222 (3.3)
Hypercoagulable state	224 (3.3)
Hypertension	3872 (57.5)
Seizure disorder	96 (1.4)
History of bleeding or thrombosis	
Bleeding	
≤30 d	166 (2.5)
>30 d	144 (2.1)
Diathesis	43 (0.6)
Prior gastrointestinal bleeding	237 (3.5)
History of embolism (not deep vein thrombosis or pulmonary embolism)	63 (0.9)
Prior deep vein thrombosis or pulmonary embolism	1145 (17.0)
Aspirin use at enrollment	
Aspirin	
≤100 mg	1441 (21.4)
>100 mg	262 (3.9)
Follow-up, mo	
Mean (SD)	16.4 (21.6)
Median (IQR)	6.7 (3.2-19.3)

Abbreviations: BMI, body mass index (calculated as weight in kilograms divided by height in meters squared); CHA_2_DS_2_-VASc, congestive heart failure, hypertension, age ≥75 years, diabetes, prior stroke or transient ischemic attack, vascular disease, age 65-74 years, sex category (female); CCI, Charlson Comorbidity Index; HAS-BLED, hypertension, abnormal kidney or liver function, stroke, bleeding history or predisposition, labile international normalized ratio, elderly, drugs or alcohol concomitantly.

^a^ Modified to exclude the labile international normalized ratio.

### Outcomes Associated With Multisite Deprescribing Intervention

**Figure 1.**
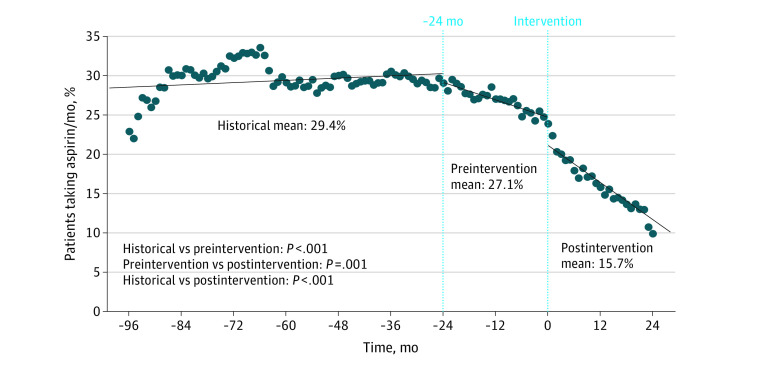
Percentage of Warfarin-Treated Patients Taking Aspirin Without an Apparent Indication by Month At baseline, 29.4% of the patient population was taking warfarin and aspirin without a history of coronary artery disease, myocardial infarction, percutaneous coronary intervention, coronary artery bypass grafting, peripheral arterial disease, heart valve replacement, use of left ventricular assist device, heart transplant, or (at some centers) a history of stroke or antiphospholipid syndrome. Starting 24 months before the intervention, a significant decrease in aspirin use was observed. After the intervention, a further significant decrease in aspirin use was achieved, with a mean postintervention rate of aspirin use of 15.7%, compared with 27.1% immediately before the intervention. *P* values compare the slopes of the regression lines.

Overall aspirin use without an indication was reduced by nearly 50% after the aspirin-deprescribing intervention, from 28.9% (95% CI, 28.4%-29.4%) before the intervention to 15.7% (95% CI, 14.7%-16.6%) after the intervention (Figure 1; eFigure 1 in the Supplement). During the historical period 1, at 96 months prior to the intervention to 24 months prior to the intervention, the percentage of patients receiving aspirin per month was unstable but generally around 30%, especially as we approached the preintervention period (at 48 months prior to the intervention, 297 of 987 [30.1%; IQR, 28.9%-31.4%]; at 36 months prior to the intervention, 312 of 1019 [30.6%; IQR, 29.4%-31.9%]) (Figure 1). Starting at 24 months prior to our intervention, the preintervention period, there was a small but significant decrease in mean aspirin use (27.1%; 95% CI, 26.1%-28.0%) compared with a mean baseline use of 29.4% (95% CI, 28.9%-29.9%); this decrease was statistically significantly stronger than during the historical period (*P* < .001 for the slope before and after 24 months before the intervention). After the intervention into the postintervention period, a significantly accelerated decrease in aspirin use was observed (mean aspirin use, 15.7%; 95% CI, 14.8%-16.5%). This decrease’s trajectory was steeper than that of the preintervention period’s trajectory (*P* = .001 for the slope before and after the intervention period) (Figure 1). Therefore, although aspirin use was decreasing prior to the intervention, an accelerated decrease was associated with the intervention.

**Figure 2.**
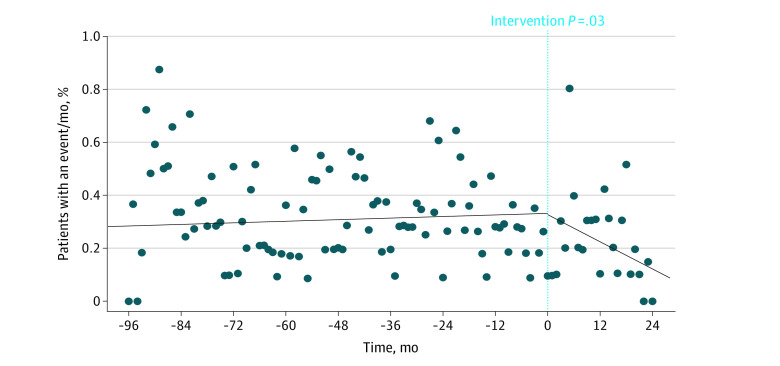
Percentage of Warfarin-Treated Patients Taking Aspirin Without an Apparent Indication by Month Who Experienced Major Bleeding There was a statistically significant decrease in major bleeding events per month during the 24 months after the intervention compared with before the intervention. *P* value compares the slopes of the regression lines.

**Figure 3.**
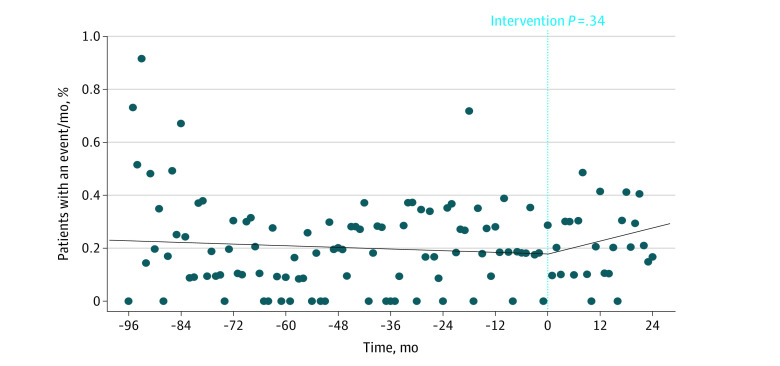
Percentage of Warfarin-Treated Patients Taking Aspirin Without an Apparent Indication by Month Who Experienced Thrombotic Events There was no statistically significant change in thrombotic events per month during the 24 months after the intervention compared with before the intervention. *P* value compares the slopes of the regression lines.

When comparing the historical and preintervention periods with the postintervention period, we observed a reduction in the mean percentage of patients with a major bleeding event (0.31% vs 0.21%; *P* = .03 for difference in slope before and after intervention; Figure 2) without a significant change in the mean percentage of patients with a thrombotic event (0.21% vs 0.24%; *P* = .34 for difference in slope before and after intervention; Figure 3). Before the intervention, a mean of 0.31% of patients (95% CI, 0.27%-0.34%) had a major bleeding event per month compared with a mean of 0.21% of patients (95% CI, 0.14%-0.28%) after the intervention (32.3% risk reduction; 1 major bleeding event prevented for every 1000 patients stopping aspirin). There was no statistically significant difference in the mean percentage of patients having any postintervention bleeding event (2.2% vs 1.3%; *P* = .12 for difference in slope before and after intervention; eFigure 2 in the Supplement), mean percentage of patients having a nonmajor bleeding event (1.9% vs 1.1%; *P* = .37 for difference in slope before and after intervention; eFigure 3 in the Supplement), mean percentage of patients having an emergency department visit for bleeding (0.94 vs 0.57; *P* = .35 for difference in slope before and after intervention; eFigure 4 in the Supplement), and mean percentage of patients admitted for bleeding (0.58% vs 0.35%; *P* = .97 for difference in slope before and after intervention; eFigure 5 in the Supplement).

### Outcomes Associated With Reduction in Aspirin Use

**Figure 4.**
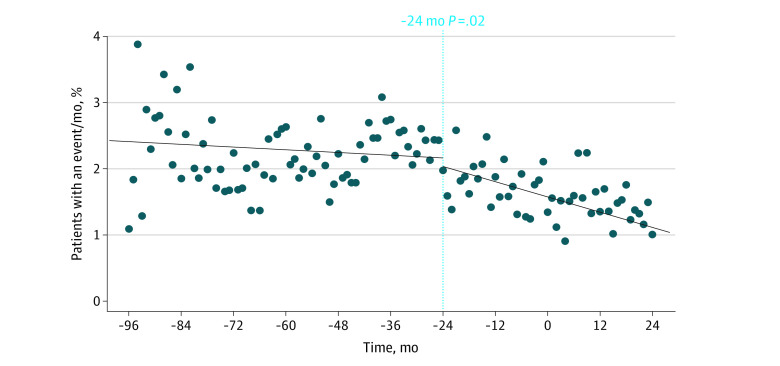
Percentage of Patients Treated With Warfarin Taking Aspirin Without an Apparent Indication by Month Who Experienced Any Bleeding There was a statistically significant decrease in bleeding events per month in the patient population before and after the observed decrease in aspirin use at 24 months before the intervention. *P* value compares the slope of the regression lines.

To evaluate the clinical association of lower aspirin use with overall outcomes, we explored clinical outcomes before aspirin use began to decrease (historical period) compared with after aspirin use began to decrease (preintervention and postintervention periods, starting 24 months before the aspirin-deprescribing intervention; Figure 1) because this was the time point when aspirin use began to decrease across all sites. Reducing aspirin use was associated with a reduction in the mean percentage of patients having a bleeding event (2.3% vs 1.5%; *P* = .02 for difference in slope before and after 24 months before the intervention; Figure 4). It was also associated with a reduction in the mean percentage of patients with a major bleeding event (0.31% vs 0.25%; *P* = .001 for difference in slope before and after 24 months before the intervention; eFigure 6 in the Supplement) and a reduction in the mean percentage of patients having an emergency department visit for bleeding (0.99% vs 0.67%; *P* = .04 for difference in slope before and after 24 months before the intervention; eFigure 7 in the Supplement). We did not observe a reduction in the mean percentage of patients with a nonmajor bleeding event (2.0% vs 1.3%; *P* = .13 for difference in slope before and after 24 months before the intervention; eFigure 8 in the Supplement) or the mean percentage of patients with an admission for bleeding (0.62% vs 0.38%; *P* = .57 for difference in slope before and after 24 months before the intervention; eFigure 9 in the Supplement). The mean percentages of patients with a thrombotic event were similar before and after reducing excess aspirin use (0.20% vs 0.23%; *P* = .36 for difference in slope before and after 24 months before the intervention; eFigure 10 in the Supplement).

## Discussion

Although inappropriate aspirin use among patients treated with warfarin has been recognized as a problem,^13^ little is known about how to reduce excess aspirin use among this high-risk population. In this multicenter, anticoagulation clinic–based quality improvement project, the intervention was associated with a significant reduction in excess aspirin use among patients treated with warfarin for atrial fibrillation and/or venous thromboembolism with no apparent indication for aspirin. After this deprescribing intervention, we observed a reduction in major bleeding events with no increase in thrombotic outcomes. Furthermore, the decrease in aspirin use that began approximately 2 years prior to our intervention was associated with a significant reduction in any bleeding, major bleeding, and emergency department visits for bleeding. These findings highlight the need for greater aspirin stewardship among patients receiving warfarin for anticoagulation. Our successful intervention across multiple health systems, with different patient populations and clinical structures, could serve as a national model for reducing excess aspirin use.

Our intervention was associated with a significant reduction in major bleeding (Figure 1). However, without a control group, it was not possible to know whether the intervention directly resulted in reduced bleeding outcomes. A previous study of our registry data found that concomitant aspirin and warfarin therapy was associated with 1 additional major bleeding event for every 36 patients receiving combination therapy instead of warfarin monotherapy.^13^ Accordingly, it could be anticipated that this intervention would have the potential for a significant clinical association with bleeding outcomes. Although event rates decreased after the intervention for all studied bleeding outcomes, we did not observe a significant postintervention reduction in any bleeding, nonmajor bleeding, or health care use (eFigures 2-5 in the Supplement). It is possible that our ability to detect a statistical difference was limited by the length of follow-up, which affected statistical power. When we look at the longer time period represented by our 24-month pre-post intervention analysis, the longer follow-up period allowed for the detection of more postintervention bleeding events and, therefore, for more statistical power (Figure 4; eFigure 6 in the Supplement).

One major challenge to a multicenter aspirin-deprescribing intervention is reaching consensus on when aspirin use is unnecessary. We focused on patient populations in which the clinicians thought there was broad agreement that concomitant use of aspirin with warfarin was generally not needed. In addition, all management decisions were left to clinicians who were directly involved in the care of the patient and able to engage in shared decision-making.

The second challenge to reducing excess aspirin use is developing a systematic approach to reducing excess aspirin use. Anticoagulation clinics are commonly used to manage the millions of patients in the US currently treated with warfarin; they are often run by physicians, pharmacists, and nurses with expertise in anticoagulation and antiplatelet therapies.^20^ An anticoagulation clinic–based intervention is a logical starting place for such an initiative to improve medication safety.^25^ The anticoagulation clinic health care professionals’ knowledge of anticoagulation therapies and their frequent interactions with patients and their physicians could facilitate meaningful changes. Although the intervention required an initial investment in time and effort, this effort demonstrated its worth through the reduction in excess aspirin use. A meticulous medication reconciliation process should be incorporated during anticoagulation clinic enrollment because this is an opportune time to address potentially unnecessary aspirin use; a previous study found that nearly one-third of patients receiving warfarin were using aspirin with warfarin despite not having a clear need for such therapy.^13^

Not all patients receiving warfarin are followed up at anticoagulation clinics. Although the expertise of the health care professionals at our anticoagulation clinics was integral to the success of this effort, this effort could readily be adapted to other settings. Specifically, a similar approach could be used by primary care physicians and subspecialty clinics to reach a broader patient population. Resources developed by the MAQI^2^ for our intervention are available online^26^ for other centers interested in adapting this work. Other centers have similarly been able to implement similar interventions^27,28^; this is one of the first studies to report on clinical outcomes, to our knowledge.

Our data show that there was a significant decrease in aspirin use about 24 months before our intervention (Figure 1). It is not clear whether this decrease was associated with prior quality improvement efforts or indirect outcomes of our preparation for this intervention. The European Society of Cardiology and the 2016 US Preventive Services Task Force guidelines on aspirin use for primary prevention^1^ immediately predated this observed decrease, and several pivotal trials on aspirin for primary prevention followed this observation.^29^ Nevertheless, with the start of our intervention, the rate of observed decrease in aspirin use significantly increased, suggesting that the intervention may be partially responsible for the improved clinical outcomes (Figure 1).

Further research is needed to determine whether deprescribing aspirin for patients receiving direct oral anticoagulants is similarly effective and to confirm our study findings, ideally with a control group. It is also unclear why so many patients treated with warfarin were receiving concomitant aspirin without a strong reason for it. In many situations, clinicians (1) may be unaware of the guidelines or data, (2) may favor antiplatelet therapy for patients with numerous or poorly controlled vascular risk factors, (3) may be unclear as to who is managing the aspirin use when multiple clinicians and subspecialists are involved (primary care, hematology, general cardiology, interventional cardiology, electrophysiology, and/or vascular surgery), and (4) may not discontinue aspirin use (often for primary prevention) with warfarin initiation. Given that aspirin is not a prescription medication, it could be postulated that clinicians may not always be aware that patients are taking aspirin, which is a barrier to aspirin-deprescribing efforts.

Although we did not directly ask patients why they were taking aspirin, we excluded many patient groups that may have been taking aspirin for secondary prevention. Accordingly, we assumed that many patients were taking aspirin for primary prevention. In fact, aspirin is used for the primary prevention of cardiovascular disease by 25% to 45% of US adults older than 40 years.^30,31,32^ A prior retrospective review^16^ suggested that aspirin was not discontinued for about 28% of patients after they developed an indication for warfarin, which seems to be the case for many of the patients in our study. The American College of Cardiology/American Heart Association guidelines^20^ and US Preventive Services Task Force guidelines^33^ no longer support aspirin use for many of these patients, even if they were not receiving concomitant warfarin.

### Limitations

Our study has several limitations. A registry-based study has inherent limitations, including the potential for missing data and the inability to infer causation without randomization. All aspirin use was per patient report, and it is possible that aspirin was discontinued and later resumed without changes in the medical record. The patients being followed up at experienced anticoagulation clinics that regularly engage in quality improvement activities may limit the generalizability of our study. Although the clinical data were collected from several diverse institutions, the study was also geographically limited to 1 state. Data on myocardial infarction were not well captured because this outcome was not the primary intent of the warfarin quality improvement registry. Patients receiving medical care for outcome events outside our hospital network may not have been well captured if they were not reported back to the anticoagulation clinic staff. Finally, the overall event rates were low, potentially limiting the statistical power of our study.

## Conclusions

Our multicenter, anticoagulation clinic–based quality improvement initiative successfully deprescribed unnecessary aspirin for patients receiving long-term warfarin therapy. Reducing aspirin use was associated with reduced bleeding outcomes without an observed increase in thrombotic outcomes. This study emphasizes the importance of appropriate aspirin stewardship for patients receiving warfarin and serves as a quality improvement deprescribing model for other health systems.

## Notes

Supplement. **eTable.** Characteristics of Aspirin Deimplementation Interventions by MAQI^2^ Location **eFigure 1.** Overlay Plot of Percent of Warfarin Treated Patients on Aspirin Without an Apparent Indication by Month and Percent of Patients Experiencing any Bleeding per Month **eFigure 2.** Percent of Warfarin Treated Patients on Aspirin Without an Apparent Indication That Experienced any Bleeding, Comparing the Historical and Preintervention Periods to the Postintervention Period **eFigure 3.** Percent of Warfarin Treated Patients on Aspirin Without an Apparent Indication That Experienced Nonmajor Bleeding, Comparing the Historical and Preintervention Periods to the Postintervention Period **eFigure 4.** Percent of Warfarin Treated Patients on Aspirin Without an Apparent Indication That Experienced Emergency Department (ED) Visits for Bleeding per Month, Comparing the Historical and Preintervention Periods to the Postintervention Period **eFigure 5.** Percent of Warfarin Treated Patients on Aspirin Without an Apparent Indication That Experienced an Admission (AD) for Bleeding, Comparing the Historical and Preintervention Periods to the Postintervention Period **eFigure 6.** Percent of Warfarin Treated Patients on Aspirin Without an Apparent Indication by Month Who Experienced Major Bleeding, Comparing the Historical Period to the Preintervention and Postintervention Periods **eFigure 7.** Percent of Warfarin Treated Patients on Aspirin Without an Apparent Indication by Month Who Experienced Emergency Department (ED) Visits for Bleeding, Comparing the Historical Period to the Preintervention and Postintervention Periods **eFigure 8.** Percent of Warfarin Treated Patients on Aspirin Without an Apparent Indication by Month Who Experienced Nonmajor Bleeding, Comparing the Historical Period to the Preintervention and Postintervention Periods **eFigure 9.** Percent of Warfarin Treated Patients on Aspirin Without an Apparent Indication by Month Who Experienced Admission (AD) for Bleeding, Comparing the Historical Period to the Preintervention and Postintervention Periods **eFigure 10.** Percent of Warfarin Treated Patients on Aspirin Without an Apparent Indication by Month Who Experienced Thrombotic Events per Month, Comparing the Historical Period to the Preintervention and Postintervention Periods
