# Supplementary material for: Assessment of an Intervention to Reduce Aspirin Prescribing for Patients Receiving Warfarin for Anticoagulation
Source: JAMA Netw Open. 2022 Sep 19;5(9):e2231973. doi: 10.1001/jamanetworkopen.2022.31973 (PMC9486454; doi:10.1001/jamanetworkopen.2022.31973)
Supplement: Supplement. — eTable. Characteristics of Aspirin Deimplementation Interventions by MAQI2 Location eFigure 1. Overlay Plot of Percent of Warfarin Treated Patients on Aspirin Without an Apparent Indication by Month and Percent of Patients Experiencing any Bleeding per Month eFigure 2. Percent of Warfarin Treated Patients on Aspirin Without an Apparent Indication That Experienced any Bleeding, Comparing the Historical and Preintervention Periods to the Postintervention Period eFigure 3. Percent of Warfarin Treated Patients on Aspirin Without an Apparent Indication That Experienced Nonmajor Bleeding, Comparing the Historical and Preintervention Periods to the Postintervention Period eFigure 4. Percent of Warfarin Treated Patients on Aspirin Without an Apparent Indication That Experienced Emergency Department (ED) Visits for Bleeding per Month, Comparing the Historical and Preintervention Periods to the Postintervention Period eFigure 5. Percent of Warfarin Treated Patients on Aspirin Without an Apparent Indication That Experienced an Admission (AD) for Bleeding, Comparing the Historical and Preintervention Periods to the Postintervention Period eFigure 6. Percent of Warfarin Treated Patients on Aspirin Without an Apparent Indication by Month Who Experienced Major Bleeding, Comparing the Historical Period to the Preintervention and Postintervention Periods eFigure 7. Percent of Warfarin Treated Patients on Aspirin Without an Apparent Indication by Month Who Experienced Emergency Department (ED) Visits for Bleeding, Comparing the Historical Period to the Preintervention and Postintervention Periods eFigure 8. Percent of Warfarin Treated Patients on Aspirin Without an Apparent Indication by Month Who Experienced Nonmajor Bleeding, Comparing the Historical Period to the Preintervention and Postintervention Periods eFigure 9. Percent of Warfarin Treated Patients on Aspirin Without an Apparent Indication by Month Who Experienced Admission (AD) for Bleeding, Comparing the Historical Period [file jamanetwopen-e2231973-s001.pdf]

Supplementary Online Content

Schaefer JK, Errickson J, Gu X, et al. Assessment of an intervention to reduce aspirin prescribing for patients receiving warfarin for anticoagulation. *JAMA Netw Open.* 2022;5(9):e2231973. doi:10.1001/jamanetworkopen.2022.31973

eTable. Characteristics of Aspirin Deimplementation Interventions by MAQI^2 Location

eFigure 1. Overlay Plot of Percent of Warfarin Treated Patients on Aspirin Without an Apparent Indication by Month and Percent of Patients Experiencing any Bleeding per Month

eFigure 2. Percent of Warfarin Treated Patients on Aspirin Without an Apparent Indication That Experienced any Bleeding, Comparing the Historical and Preintervention Periods to the Postintervention Period

eFigure 3. Percent of Warfarin Treated Patients on Aspirin Without an Apparent Indication That Experienced Nonmajor Bleeding, Comparing the Historical and Preintervention Periods to the Postintervention Period

eFigure 4. Percent of Warfarin Treated Patients on Aspirin Without an Apparent Indication That Experienced Emergency Department (ED) Visits for Bleeding per Month, Comparing the Historical and Preintervention Periods to the Postintervention Period

eFigure 5. Percent of Warfarin Treated Patients on Aspirin Without an Apparent Indication That Experienced an Admission (AD) for Bleeding, Comparing the Historical and Preintervention Periods to the Postintervention Period

eFigure 6. Percent of Warfarin Treated Patients on Aspirin Without an Apparent Indication by Month Who Experienced Major Bleeding, Comparing the Historical Period to the Preintervention and Postintervention Periods

eFigure 7. Percent of Warfarin Treated Patients on Aspirin Without an Apparent Indication by Month Who Experienced Emergency Department (ED) Visits for Bleeding, Comparing the Historical Period to the Preintervention and Postintervention Periods

eFigure 8. Percent of Warfarin Treated Patients on Aspirin Without an Apparent Indication by Month Who Experienced Nonmajor Bleeding, Comparing the Historical Period to the Preintervention and Postintervention Periods

eFigure 9. Percent of Warfarin Treated Patients on Aspirin Without an Apparent Indication by Month Who Experienced Admission (AD) for Bleeding, Comparing the Historical Period to the Preintervention and Postintervention Periods

eFigure 10. Percent of Warfarin Treated Patients on Aspirin Without an Apparent Indication by Month Who Experienced Thrombotic Events per Month, Comparing the Historical Period to the Preintervention and Postintervention Periods

This supplementary material has been provided by the authors to give readers additional information about their work.

© 2022 Schaefer JK et al. *JAMA Network Open.*

**eTable.** Characteristics of Aspirin Deimplementation Interventions by MAQI[2] Location			
Location[a]	Census	Added exclusions	Select Intervention Characteristics
Site A<br>Urban	5,000 patients	History of stroke	--Medical residents review an EMR generated report of patients on warfarin+ASA. For patients with an UI-ASA, the PCP is contacted. If the provider opts to discontinue ASA, a note is generated in the patient's chart. The anticoagulation clinic then contacts the patient to suggest ASA is discontinued.<br>--For new patients referred to the anticoagulation clinic, pharmacists review all medications and will contact the provider when there is an UI-ASA. If ASA is to be discontinued per the provider recommendation, the pharmacist will put a note in the patient's chart and the anticoagulation clinic provider will contact the patient to suggest ASAis stopped.
Site B<br>Urban	300 patients	None	--Anticoagulation clinic patients are periodically, manually reviewed by providers to identify if there isan UI-ASA or I-ASA<br>--Providers and/or subspecialists are contacted to clarify the need for ASA.
Site C<br>Urban	3,000 patients	None	--Patients are asked if they are taking ASA. If there is an UI-ASA, the ongoing use of ASA is reviewed by a clinician who determines if further action is needed.
Site D<br>Rural	450 patients	None	--ASA use is reviewed at each follow-up visit using an assessment form, along with co-morbidities that may suggest a need for warfarin+ASA. If there is an UI-ASA or I-ASA is identified, a letter is faxed to the provider and a note is generated in the patient chart. If the provider does not respond by the next patient follow-up visit, another attempt is made to contact the provider. If the provider declines to discontinue ASA, this is documented.
Site E<br>Urban	2,200 patients	Stroke, APLS	--Patients are periodically screened for an UI-ASA or I-ASA by the anticoagulation clinic staff. The providers of patients identified as having an UI-ASA or I-ASA are contacted (letter is faxed to provider or sent through the EMR) to determine if ASA should be discontinued. If the provider does not respond, two additional attempts are made to contact the provider (letter sent followed by phone call to the provider's office).
Site F<br>Urban	4,000 patients	Stroke, APLS, heart transplant	--A report is generated of patients on warfarin+ASA with an UI-ASA or I-ASA. Anticoagulation clinic nurses contact patients regarding their ASA usage and review the indication for combination therapy.Letters are sent through the EMR to providers to determine if there is an ongoing need for ASA.<br>--There is a plan to add a "flag" to the medical record to identify patients that need to be reviewed.

[a]Location rural/urban status determined by rural-urban commuting area code of the primary affiliated hospital.

Abbreviations: ASA, acetylsalicylic acid or aspirin; APLS, antiphospholipid antibody syndrome; I-ASA, inappropriate aspirin use; EMR, electronic medical record; MAQI[2], Michigan Anticoagulation Quality Improvement Initiative; PCP, primary care provider; UI-ASA, unclear indication for aspirin use.

© 2022 Schaefer JK et al. *JAMA Network Open.*

eFigure 1. Overlay Plot of Percent of Warfarin Treated Patients on Aspirin Without an Apparent Indication by Month and Percent of Patients Experiencing any Bleeding per Month

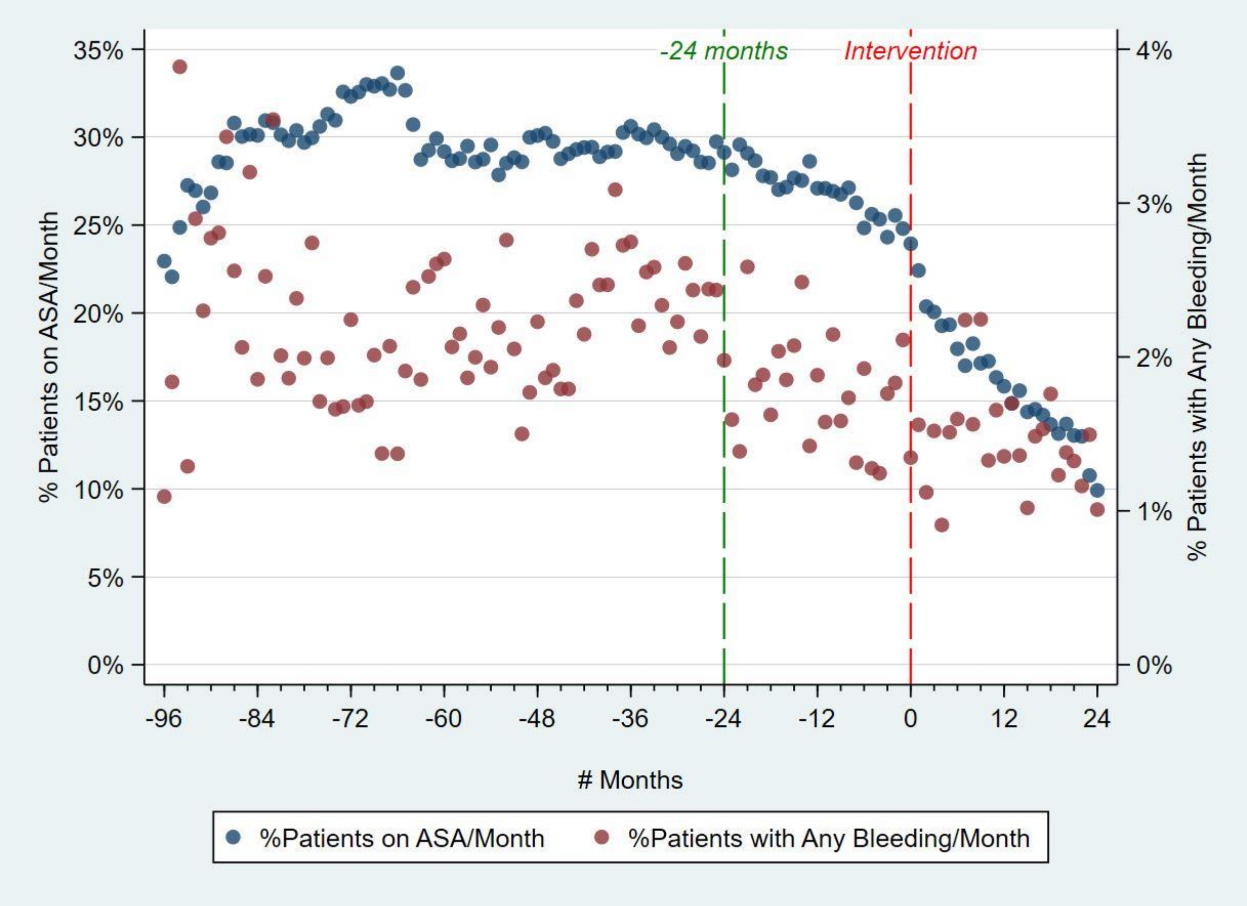

Caption: The blue dots represent the percentage of patients on warfarin and aspirin as assessed at monthly intervals. The red dots represent the percentage of patients who experienced a bleeding event per month, similarly assessed at monthly intervals, with an overall decline over time.

© 2022 Schaefer JK et al. *JAMA Network Open.*

eFigure 2. Percent of Warfarin Treated Patients on Aspirin Without an Apparent Indication That Experienced any Bleeding, Comparing the Historical and Preintervention Periods to the Postintervention Period

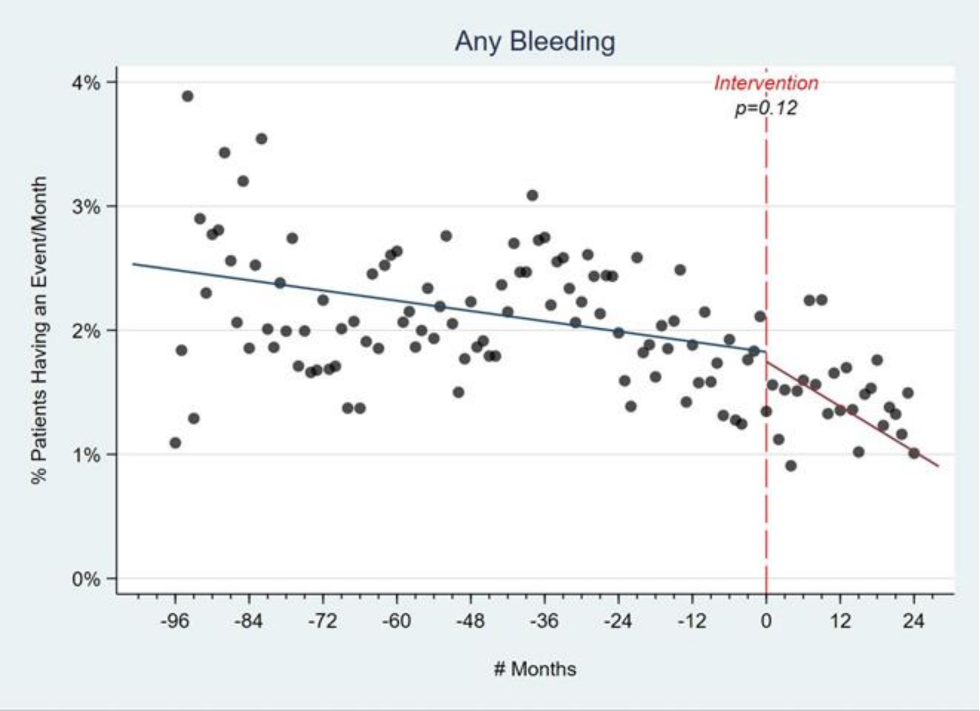

Caption: We did not observe a statistically significant decline in bleeding events per month in our patient population before and after the intervention. P-value compares the slope of the regression lines.

© 2022 Schaefer JK et al. *JAMA Network Open.*

eFigure 3. Percent of Warfarin Treated Patients on Aspirin Without an Apparent Indication That Experienced Nonmajor Bleeding, Comparing the Historical and Preintervention Periods to the Postintervention Period

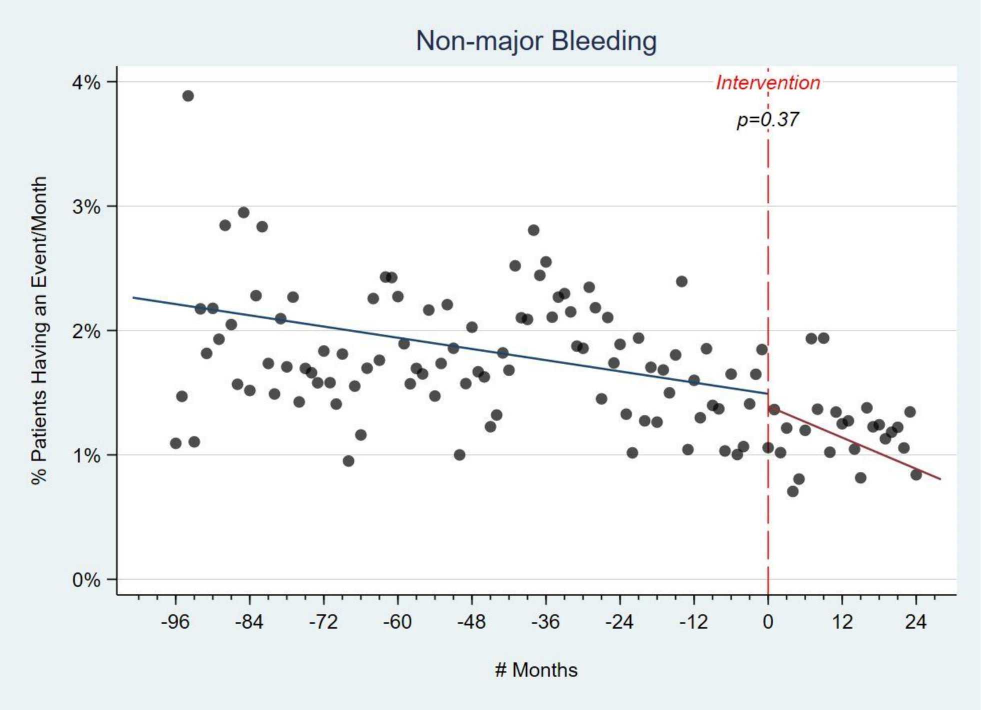

Caption: We did not observe a statistically significant decline in non-major bleeding events per month in our patient population before and after the intervention. P-value compares the slope of the regression lines.

© 2022 Schaefer JK et al. *JAMA Network Open.*

eFigure 4. Percent of Warfarin Treated Patients on Aspirin Without an Apparent Indication That Experienced Emergency Department (ED) Visits for Bleeding per Month, Comparing the Historical and Preintervention Periods to the Postintervention Period

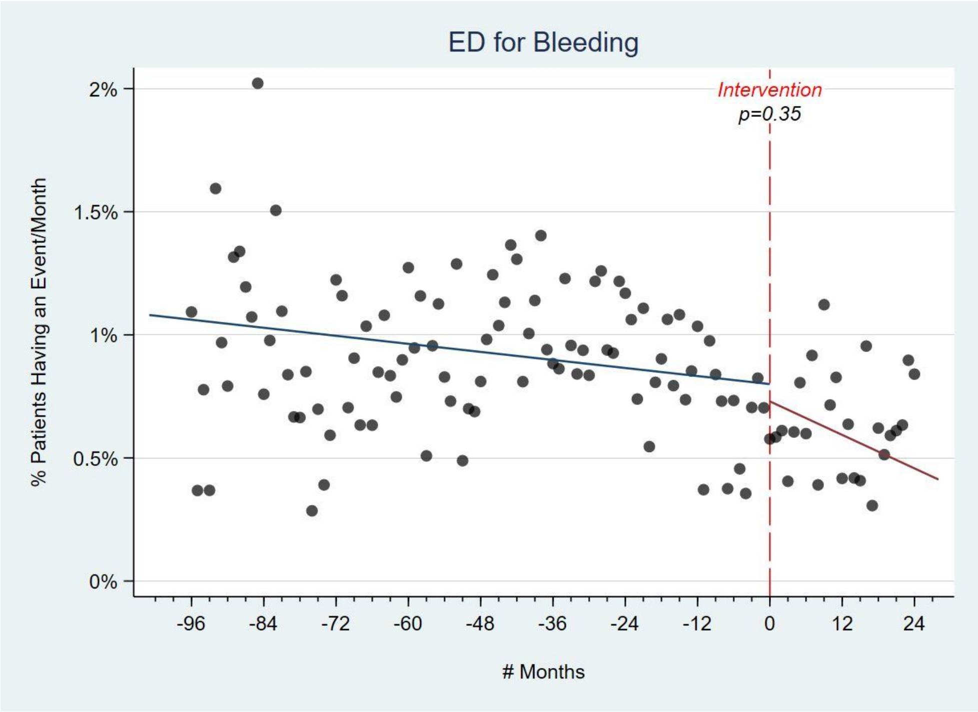

Caption: We did not observe a statistically significant decline in ED visits per month in our patient population before and after the intervention. P-value compares the slope of the regression lines.

© 2022 Schaefer JK et al. *JAMA Network Open.*

eFigure 5. Percent of Warfarin Treated Patients on Aspirin Without an Apparent Indication That Experienced an Admission (AD) for Bleeding, Comparing the Historical and Preintervention Periods to the Postintervention Period

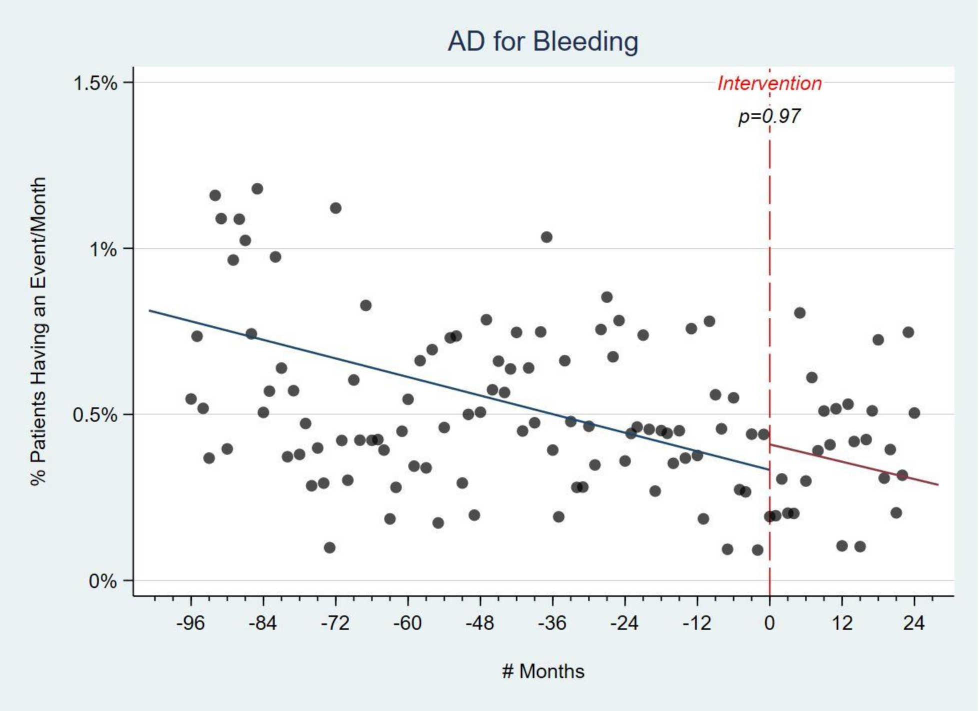

Caption: We did not observe a statistically significant decline in admissions for bleeding per month in our patient population before and after the intervention. P-value compares the slope of the regression lines.

© 2022 Schaefer JK et al. *JAMA Network Open.*

eFigure 6. Percent of Warfarin Treated Patients on Aspirin Without an Apparent Indication by Month Who Experienced Major Bleeding, Comparing the Historical Period to the Preintervention and Postintervention Periods

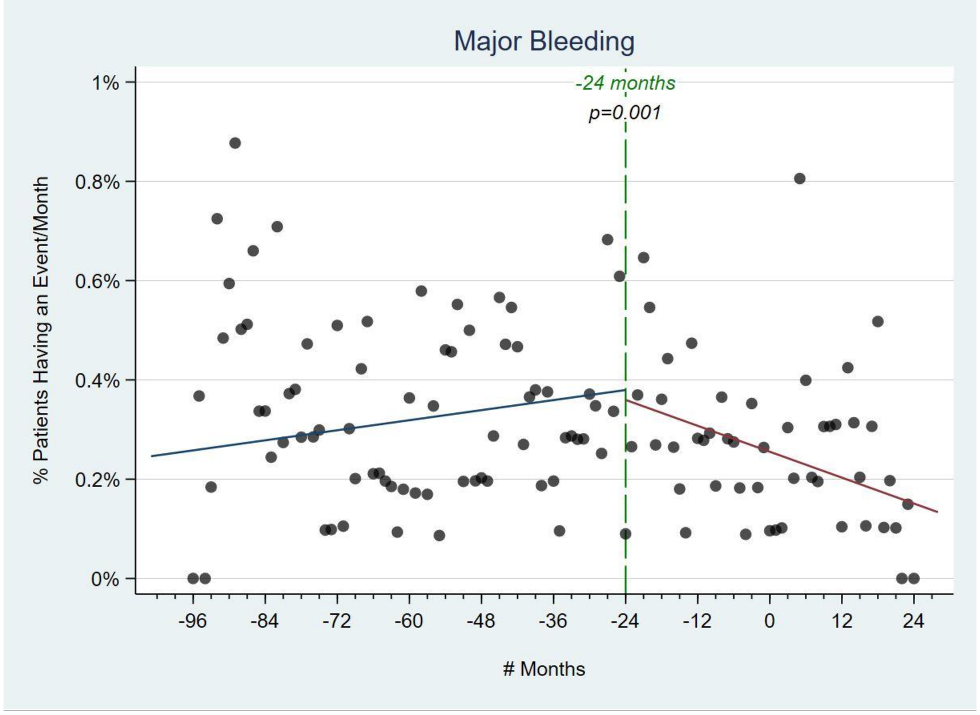

Caption: We observed a statistically significant decline in major bleeding events per month in our patient population before and after the observed decline in aspirin use at 24 months pre-intervention. P-value compares the slope of the regression lines.

© 2022 Schaefer JK et al. *JAMA Network Open.*

eFigure 7. Percent of Warfarin Treated Patients on Aspirin Without an Apparent Indication by Month Who Experienced Emergency Department (ED) Visits for Bleeding, Comparing the Historical Period to the Preintervention and Postintervention Periods

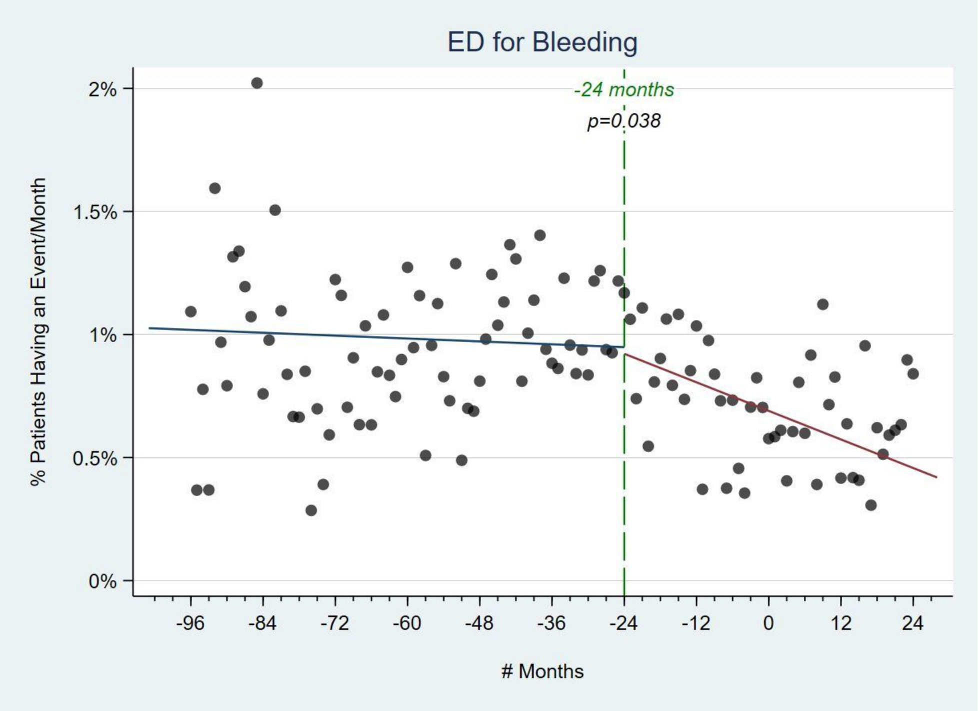

Caption: We observed a statistically significant decline in ED visits for bleeding per month in our patient population before and after the observed decline in aspirin use at 24 months pre-intervention. P-value compares the slope of the regression lines.

© 2022 Schaefer JK et al. *JAMA Network Open.*

eFigure 8. Percent of Warfarin Treated Patients on Aspirin Without an Apparent Indication by Month Who Experienced Nonmajor Bleeding, Comparing the Historical Period to the Preintervention and Postintervention Periods

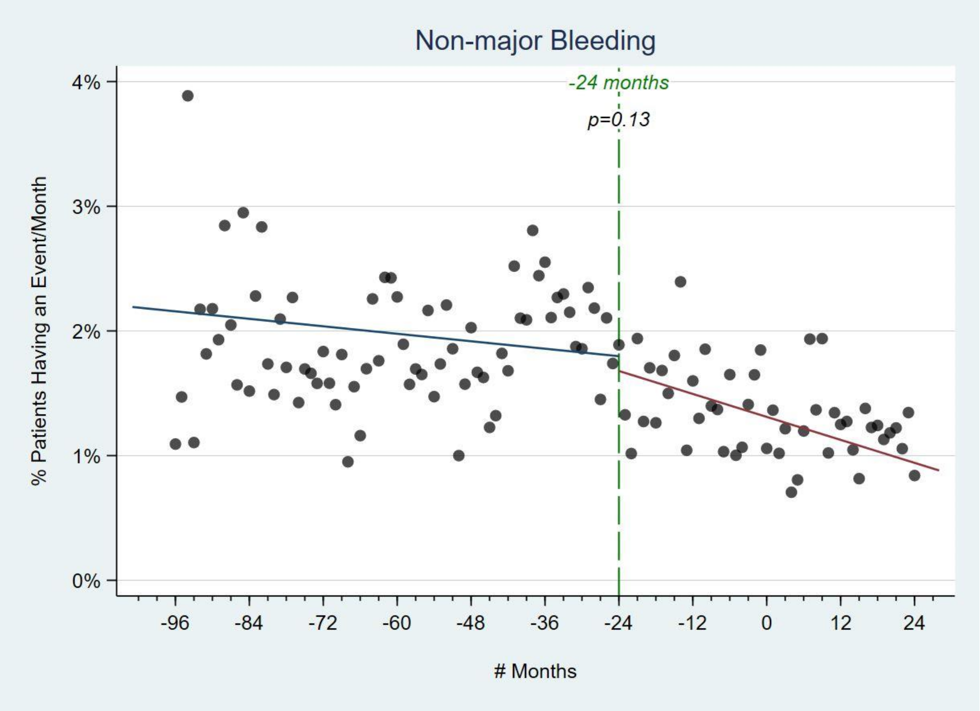

Caption: We observed no significant change in non-major bleeding events per month in our patient population before and after the observed decline in aspirin use at 24 months pre-intervention. P-value compares the slope of the regression lines.

eFigure 9. Percent of Warfarin Treated Patients on Aspirin Without an Apparent Indication by Month Who Experienced Admission (AD) for Bleeding, Comparing the Historical Period to the Preintervention and Postintervention Periods

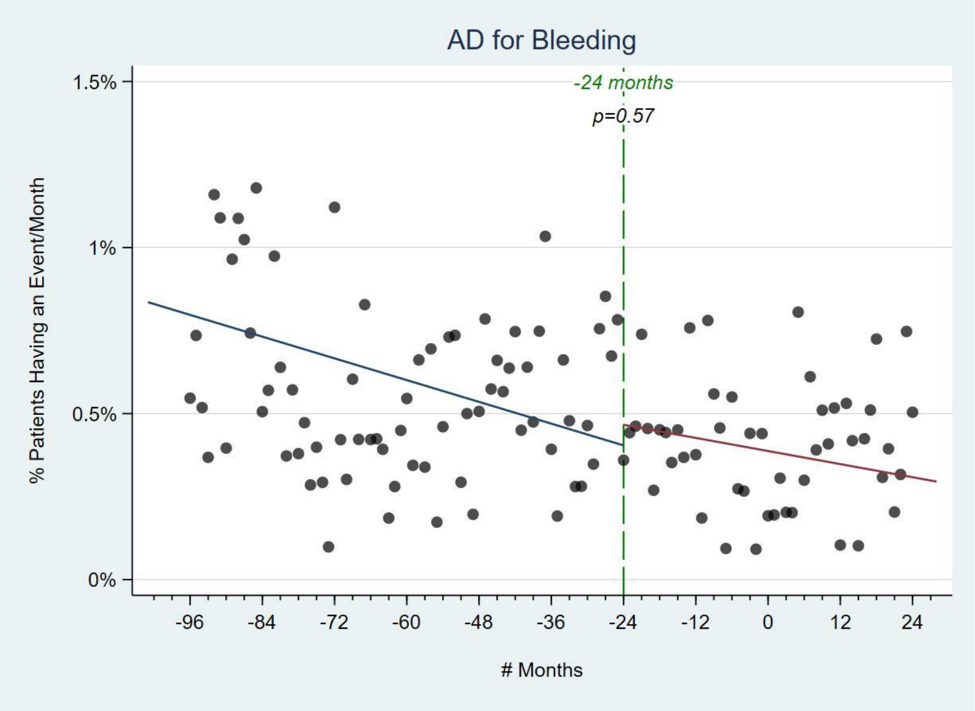

Caption: We observed no significant change in admissions for bleeding per month in our patient population before and after the observed decline in aspirin use at 24 months pre-intervention. P-value compares the slope of the regression lines.

© 2022 Schaefer JK et al. *JAMA Network Open.*

eFigure 10. Percent of Warfarin Treated Patients on Aspirin Without an Apparent Indication by Month Who Experienced Thrombotic Events per Month, Comparing the Historical Period to the Preintervention and Postintervention Periods

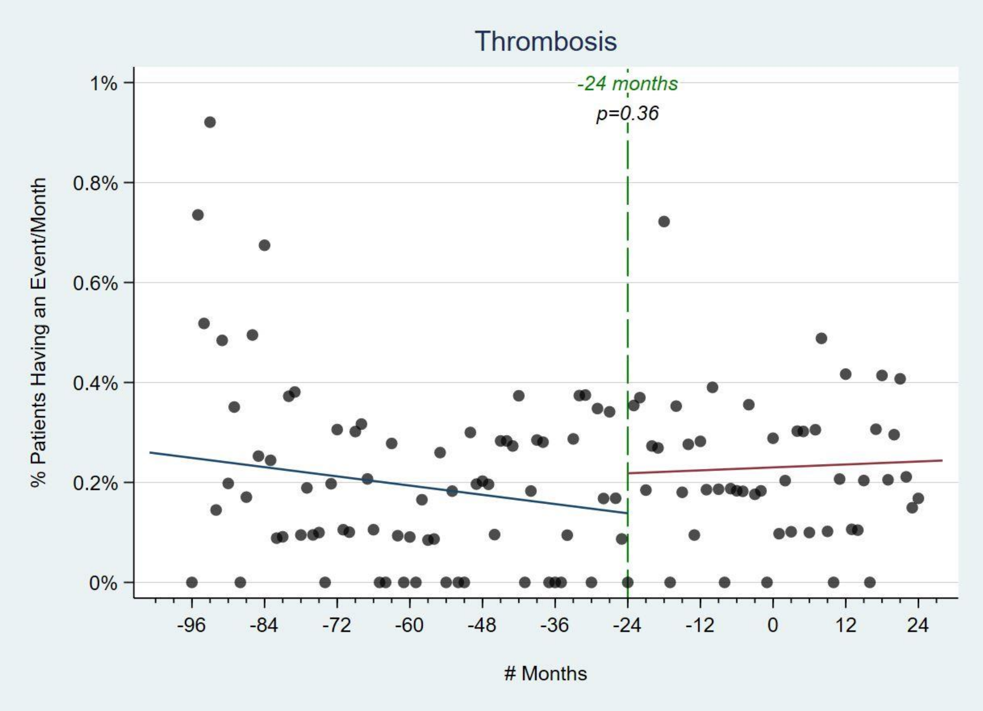

Caption: We observed no significant change in thrombotic events per month in our patient population before and after the observed decline in aspirin use at 24 months pre-intervention. P-value compares the slope of the regression lines.

© 2022 Schaefer JK et al. *JAMA Network Open.*
